# Effects of Tempeh Probiotics on Elderly With Cognitive Impairment

**DOI:** 10.3389/fnagi.2022.891773

**Published:** 2022-06-24

**Authors:** Yvonne Suzy Handajani, Yuda Turana, Yogiara Yogiara, Sagita Pratiwi Sugiyono, Vincent Lamadong, Nelly Tina Widjaja, Geovannie Audrey Moniqe Christianto, Antonius Suwanto

**Affiliations:** ^1^Center of Health Research, Atma Jaya Catholic University of Indonesia, Jakarta, Indonesia; ^2^Department of Neurology, School of Medicine and Health Sciences, Atma Jaya Catholic University of Indonesia, Jakarta, Indonesia; ^3^Faculty of Biotechnology, Atma Jaya Catholic University of Indonesia, Jakarta, Indonesia; ^4^School of Medicine and Health Sciences, Atma Jaya Catholic University of Indonesia, Jakarta, Indonesia; ^5^Department of Biology, Faculty of Mathematics and Natural Science, IPB University, Bogor, Indonesia

**Keywords:** cognitive, elderly, *Limosilactobacillus fermentum*, probiotic, tempeh

## Abstract

**Introduction:**

Oral consumption of probiotics can alter Gut Microbiota by causing changes in the production of probiotic derivatives. Therefore, by utilizing Gut-Brain-Axis (GBA), probiotics could provide an opportunity for central nervous system (CNS) modulation, including cognitive function. Tempeh is a traditional Indonesian food rich in probiotics and beneficial for cognitive function. However, the type of probiotics that play a role in cognitive improvement and the number of probiotics needed for the benefits of increasing cognitive function was unknown.

**Method:**

This experimental study involved a total of 93 subjects, divided into 3 groups: A, B and C/control (n: 33, 32, and 28), who were provided with probiotic supplementation isolated from tempeh for 12 weeks intervention. Inclusion criteria were age > 60 years, and memory impairment with the third repetition value of Word List Memory Immediate Recall (WLMIR) < 7. Subjects with diabetes were excluded. Cognitive function examinations were carried out before and after treatment. The tempeh-derived probiotics were prepared trough several processes. Genomic isolation, detection of GABA-encoding genes, and species identification using the 16S-rRNA gene encoding were performed.

**Results:**

The probiotics isolate used in the intervention was identified as *Limosilactobacillus fermentum*. We assigned this isolate as *L. fermentum* A2.8. The presence of the gene encoding GABA was found on this isolate. There was an increase in the cognitive domains of memory, learning process, and verbal fluency (*p* < 0.05) in group A (probiotics at concentration of 10^8^ CFU/mL). Memory function, visuospatial, and verbal fluency improved (*p* < 0.05) in group B (probiotics at concentration of 10^7^ CFU/mL). Only an increase in the memory domain was observed in the control group. Improvement of the learning process occurred only in group A (*p* = 0.006).

**Conclusion:**

Administration of probiotics derived from *L. fermentum A2.8* increased the cognitive domains of memory, language and visuospatial function. However, probiotic supplementation at a concentration of 10^8^ CFU/mL was better in improving the learning process. This study succeeded in detecting Lactic Acid Bacterial isolates *L. fermentum A2.8* that enclosed gene encoding glutamate decarboxylase (gad) which is involved in the synthesis of -aminobutyric acid (GABA), a neurotransmitter vital for cognitive function.

## Introduction

With the increase in life expectancy, health issues associated with aging must be addressed. Currently, cognitive impairment is a common health problem found among the elderly. It is estimated that people with dementia will continue to increase in the future, especially in Asia (Nichols et al., 2022).

Studies have demonstrated that microbiota performs a variety of roles in brain health, with the Gut-Brain Axis (GBA) showing a close relationship between the digestive system and the brain (Carabotti et al., 2015). Various studies have demonstrated the mechanisms underlying the GBA. The presence and diversity of microbiota in the gut greatly influences the GBA mechanism, ultimately affecting cognitive function and signifying that cognitive function does not merely rely on an internal relationship through neuronal mechanisms. The gut microbiota (GM) influences both brain structure and cognition (Carabotti et al., 2015; Fernandez-Real et al., 2015; Chen et al., 2021; Feng et al., 2022). Modifying gut probiotic composition by probiotic supplementation might contribute to a prevention and therapy methods of Alzheimer's Disease (AD) (Kowalski and Mulak, 2019).

Oral consumption of probiotics can alter GM by increasing the diversity and number of beneficial microbes, causing changes in the production of probiotic derivatives, reducing inflammation, changing HPA axis function, and altering gut barrier integrity (Plaza-Diaz et al., 2019). Therefore, by utilizing GBA, probiotics could provide an opportunity for central nervous system (CNS) modulation and serve as a possible therapeutic adjunct for some CNS-related conditions (Genedi et al., 2019). Wang et al. (2020) and Yang et al. (2020) also stated that probiotics could change gut dysbiosis and microbiota, improve cognitive function decline, decrease Aβ levels in the hippocampus (associated with the pathophysiology of AD), maintain neuronal structural integrity and plasticity, and reduce trimethylamine-n-oxide (TMAO) synthesis and neuroinflammation. Park et al. (2020) showed that *L. fermentum* exerts a beneficial effect on the regulation of immune response and can provide health improvements, including cognition. Other study has stated that *L*. fermentum produce neuromodulator which contributes to improve cognitive function by inhibiting acetylcholinesterase (AChE) activity (Musa et al., 2017).

Tempeh is a traditional Indonesian food rich in probiotics and beneficial for cognitive function (Stéphanie et al., 2017; Stephanie et al., 2018). A study by Handajani et al. (2020) found that the intervention of giving 100 grams of tempeh improved cognitive function in the elderly compared to controls. However, in these studies, the type of probiotics that play a role in cognitive improvement and the number of probiotics needed for the benefits of increasing cognitive function was unknown.

This study aimed to determine how beneficial the administration of probiotics derived from tempeh is for the elderly with cognitive impairment. The study was done in two stages: first, by isolating the type of bacteria that is safe and beneficial, then by providing those probiotics in two different concentrations to the elderly with cognitive impairment.

## Materials and Methods

This study was an experimental study, which included two phases: the preparation of isolates, and provision of the intervention isolates.

### Intervention Isolate Preparation

#### Bacterial Isolation

Lactic acid bacteria (LAB) used in this study were isolated from tempeh A, which were previously studied to have the ability to improve cognitive functions among the elderly (Handajani et al., 2020). The LABs were grown and maintained on de Mann Rogosa and Sharpe Agar media. (MRSA) (Oxoid, Oxoid, Ltd., Hampshire, UK) supplemented with 0.3% CaCO_3_ (Chen et al., 2010). They were then incubated under anaerobic conditions, with an incubation temperature of 35°C for 24–48 h. Gram assays and catalase tests confirmed the grown colonies. Hemolysis tests were also carried out for the initial safety screening of the bacterial isolates. The hemolysis tests were performed on Blood Agar Base media (Oxoid, Oxoid, Ltd., Hampshire, UK). We routinely culture the isolate in MRS media which is the standard medium to grow LAB (Onda et al., 2002; Chen et al., 2010; Hwanhlem et al., 2011).

#### Genomic Isolation, Detection of GABA-Encoding Genes, and Identification Using the 16S-rRNA Encoding Gene

Genomic DNA was extracted using the Wizard® Genomic DNA Purification Kit (Promega, Corp., Madison, United States). The isolation procedure followed the protocol described in the manual kit. The glutamate decarboxylase gene (*gad*) were detected through the PCR amplification technique (Lin et al., 2017; Wu et al., 2017). Glutamate decarboxylase is the enzyme involved in gamma-aminobutyric acid (GABA) synthesis. The primers used to amplify the *gad* gene (Table 1) is a modification of the primers listed by Wu et al. (2017) by eliminating the restriction enzyme recognition sequence present in the primary sequence. The positive isolates for the *gad* gene were then identified using DNA sequencing of the *pheS* encoding gene (Archer and Halami, 2015). The pheS coding gene was amplified using the pheS-F primer (5′-CGCCAGACATCTTCAAGACG- 3′) and pheS-R (5′-GAGCGGCTGGAAGAATTACG-3′) (Archer and Halami, 2015). The isolates used for the intervention were reconfirmed by their species using DNA sequencing of the gene encoding 16S-rRNA. For identification, primers 63F (5′-CAGGCCTAACACATGCAAGTC-3′) and 1387R (5′-GGGCGGWGTGTACAAGGC-3′) were used (Marchesi et al., 1998). The PCR conditions followed the steps described by Marchesi et al. (1998) DNA Sequencing was carried out at Apical Scientific Sdn. Bhd, Malaysia.

**Table 1:** Primary sequences for *gad* gene detection.

**Primary**	**Nucleotide sequence primer (5^′^−3^′^)**	**References**
Lb-gadA-F	ATGAATAAAAACGATCAGGAAAC	Wu et al., 2017
Lb-gadA-R	TTAACTTCGAACGGTGGTC	
Lb-gadB-F	ATGGCTATGTTGTATGG	
Lb-gadB-R	TTAGTGCGTGAACCCGTATT	
Lp-gadB-F	ATGGCAATGTTATACGGTAAACAC	
Lp-gadB-R	TCAGTGTGTGAATCCGTATTTC	
gadBFerm-1	ATGTCACTTTACGGAAAGTACGACCAAG	Lin et al., 2017
gadBFerm-2	TTAGTGGGTAAAGCCGTACTTTTTCAGG	

#### Bacterial Microencapsulation Using Maltodextrin

Isolates used for the microencapsulation process were randomly selected and based on the detection of the *gad* gene. For the microencapsulation of bacteria, *L. fermentum* A2.8 was used with two concentrations of bacterial cells, 10^8^ and 10^7^ CFU/mL, respectively. The microencapsulation process used maltodextrin DE 10–12 (Qinhuangdao Lihuastarch Co. Ltd., China) as filler. The cell suspension was mixed with 10% Maltodextrin DE 10–12. As a control in the intervention experiment, 10% Maltodextrin without cell suspension was used. Microencapsulation was done by spray-drying (Mini Spray Dryer B-290, BÜCHI Labortechnik AG, Flawil, Switzerland). The microencapsulation process followed the procedure described by Bhagwat et al. (2020) with modifications to the spray-dry conditions, which were as follows; inlet temperature 120°C, aspirator 95%, pump 18%, flow 45 ml/min, and outlet temperature 68°C. All microbiological processes, including bacteria culture and microencapsulation, was performed in the laboratory at the Faculty of Biotechnology Atma Jaya Catholic University of Indonesia, Indonesia.

### Preparation of Subjects for Intervention

The participants were recruited in the elderly community which was part of the elderly target area of School of Medicine and Health Sciences, Atma Jaya Catholic University of Indonesia. The inclusion criteria were elderly with memory disorders with the value of the third repetition of Word List Memory Immediate Recall (WLMIR) < 7 (Fillenbaum et al., 2008; Turana et al., 2014). The exclusion criteria were a diagnosis of diabetes or blood glucose of > 200 mg/dL, to avoid an increase in blood glucose level, since the extract given were mixed with artificial flavoring, severe vision and hearing impairments that inhibited the interview process. Participants who were conducted in this study were not living alone and accompanied with family members as their caregiver during consent and interview.

**Figure 1:**
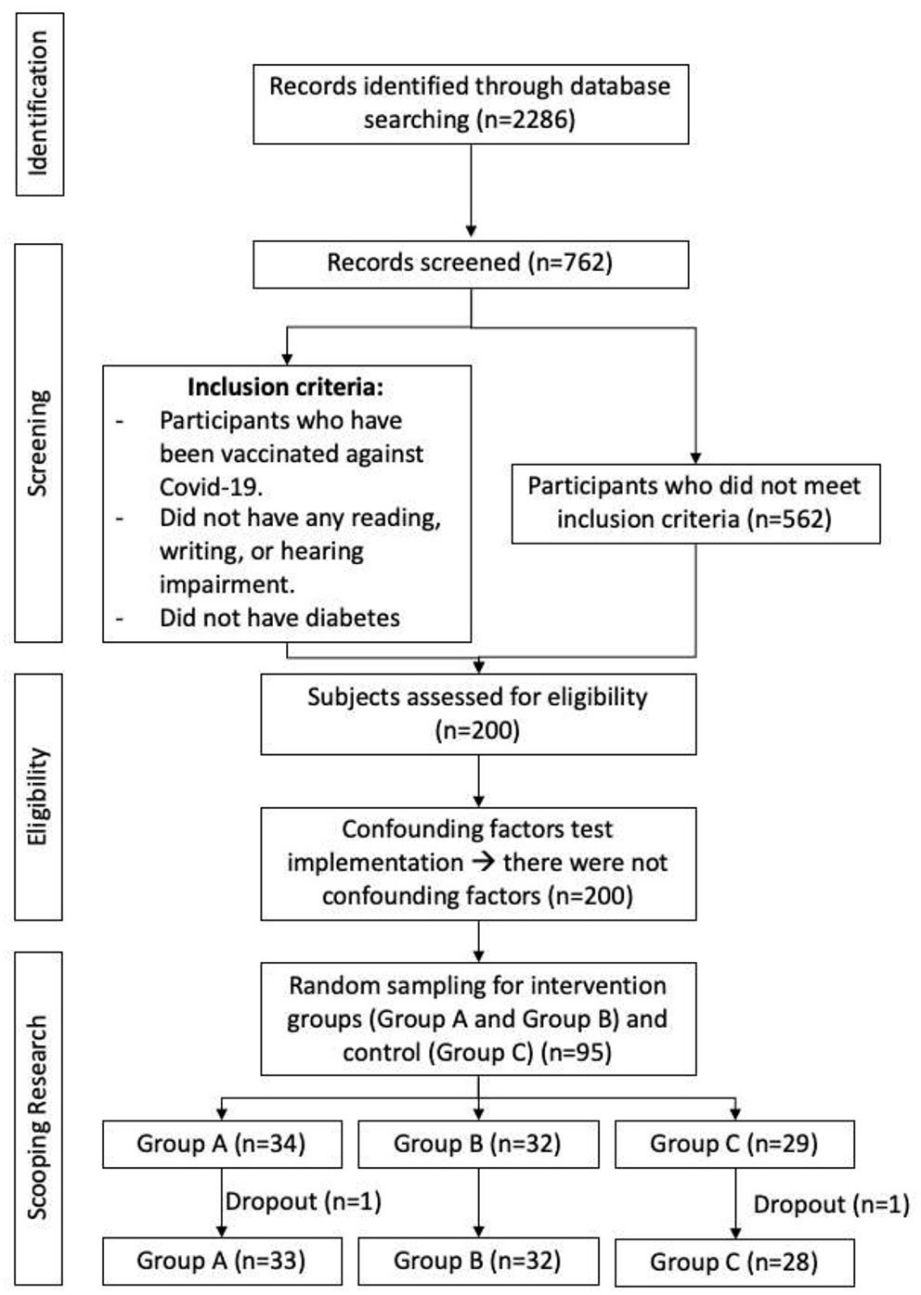
Consort flow chart of participants recruitment.

A total of 200 subjects were eligible and examined for physical health and cognitive function as well as other health variables, such as diabetes, hypertension, dyslipidemia, heart disease, lung disease, joint disorders, depression, eye, and ear disorder. With random sampling without blinding, the intervention phase was conducted on three groups of elderly: group A, B, and Control, each with 33, 32, 28 subjects, respectively, with a total of 93 participants (Figure 1). Cognitive function assessments were carried out on subjects who met the criteria before and after the intervention. The cognitive function domains assessed were memory (WLMIR) and language function (verbal fluency) using tools from the Consortium to Establish a Registry for Alzheimer's Disease (CERAD), and visuospatial function using the Clock Drawing Test (CDT) assessment (Fillenbaum et al., 2008; Kim et al., 2018). Cognitive tests were performed by doctors (SPS, VL) who have received training and were supervised by a neurologist (YT).

The interventions provided to the three subject groups were: intervention A probiotic powder with a cell density equivalent to OD1, intervention B probiotic powder with 10 times more dilute *Lactobacillus* bacteria, and maltodextrin powder for the control subjects. The probiotic intervention was given as a pre-packaged solution in bottles with 100 ml water solvent and orange-flavoring. This research was conducted for 12 weeks. Participants were urged to stop consuming any fermented-derived foods (such as tofu, soy milk, yogurt, and cassava tape). Control group was not given any probiotics. Trained workers who have been briefed, distributed probiotics to participants as well as monitoring and evaluating daily compliance of consumption using a card. This card functions like both a calendar and reminder to the participants and trained workers.

Univariate analysis was conducted to see the demographic characteristics of the respondents. Then a bivariate analysis was performed using the Wilcoxon matched-pairs test to compare the pre and post-intervention values on cognitive variables. BM SPSS software version 22 (IBM, New York, USA) was used for statistical analyses, with statistical significance set at p < 0.05. This research was approved by the ethics committee of Atma Jaya Catholic University of Indonesia FKIK NO: 10/07/KEP/-FKUAJ/2019 and received approval from each subject.

## Results

### Lactic Acid Bacteria Isolate

There were bacteria colonies identified as lactic acid bacteria during the bacteria separation process, which generated a distinct, clear zone surrounding the colonies. This clear zone appears due to the acid produced by bacteria dissolving the CaCO_3_ in the media. After identifying the lactic acid bacteria, 24 isolates were randomly picked from Tempeh A. The selection of these isolates was made subjectively based on the morphology of the growing colonies.

The isolates that did not produce a clear zone were confirmed as Gram-positive bacteria and catalase-negative. All bacterial isolates did not have hemolytic activity. Meanwhile, three isolates, namely isolate A2.7, A2.8, and A4.9 were detected to have the *gadB* gene after amplification using gadBFerm-1 and gadBFerm-2 primer pair. Detection using other primer pairs did not show positive results. Identification using the *pheS* gene on the three isolates showed similarities to the bacterium *Limosilactobacillus fermentum (L. fermentum)*. Among the three isolates, isolate A2.8 was randomly selected to be microencapsulated into probiotic powder for intervention purposes. To assure its identity, isolate A2.8 was re-identified using the 16S-rRNA gene, and the results consistently showed similarities (99.7%) to *L. fermentum*. Hence, we assigned isolate A2.8 as *L. fermentum* A2.8. The microencapsulation process produced three types of probiotic powder: group A probiotic powder containing 10^8^ CFU/mL probiotic, group B containing 10^7^ CFU/mL probiotic, and group C containing only maltodextrin powder. To determine the presence or absence of contamination, a check was carried out on Eosine Methylene Blue Agar media to detect Enterobacteriaceae group and the results were zero (not detected).

We made the orange-flavored probiotics drink by mixing the microencapsulated powder with 100 ml of orange-flavored solution, and no food preservation was added to this solution. Each subject received seven bottles of this solutions. These drinks were distributed to the subjects every week and suggested to be stored in the refrigerator before use. However, the viability test of probiotics after solubilization in this solution has not been conducted and might become a limitation of this study because bacterial viability during storage could not be assured.

### Intervention on Subjects

During the study, 1 subject dropped out from group A and 1 subject from the Control group- ultimately, the number of subjects who completed research in each group was not significantly different. Most of the study participants were aged 65 years and over (77.4%), female (64.5%), and had an educational background below 9 years (84.9%) (Table 3). Table 2 also shows no significant difference between the three study groups in sociodemographic aspects, with an exception in group B where the percentage of women is higher than the other groups. The reasons for dropping out of the study were tedium in taking daily supplements and changing places of residence. There were not any confounding factors such as diet and medications.

**Table 2:** Baseline cognitive scores of group A, B, and Control.

**Variable**	**N total (%) = 93**	**Group A (*n =* 33)**		**Group B (*n =* 32)**		**Control (*n =* 28)**		***p* value**
		**Mean**	**Min-Max (Median)**	**Mean**	**Min-Max (Median)**	**Mean**	**Min-Max (Median)**	
CDT	93 (100%)	0.61	0–2 (0)	0.69	0–2 (0)	0.86	0–2 (0)	0.589
WLMIR 1	93 (100%)	2.21	0–5 (2)	2.31	0–6 (2)	2.64	0–5 (3)	0.543
WLMIR 3	93 (100%)	3.88	0–8 (4)	4.63	0–8 (5)	5.18	3–9 (5)	0.060
Learning process	93 (100%)	1.67	−2–4 (2)	2.31	−3–6 (2)	2.54	0–5 (3)	0.141
Verbal fluency	93 (100%)	9.97	5–18 (10)	11.47	4–24 (11)	11.71	3–20 (12)	0.309

**Table 3:** Demographic characteristics of group A, B, and Control.

**Variable**		**N total (%) = 93**	**Group A**	**Group B**	**Control**	***p* value**
**Demographic characteristics**						
Age	≥65	72 (77.4)	26 (36.1)	25 (34.7)	21 (29.2)	0.934
	60–65	21 (22.6)	7 (33.3)	7 (33.3)	7 (33.3)	
Gender	Female	60 (64.5)	23 (38.3)	18 (30)	19 (31.7)	0.026
	Male	33 (35.5)	10 (30.3)	14 (42.4)	9 (27.3)	
Education	<9 years	79 (84.9)	29 (36.7)	23 (29.1)	27 (34.2)	0.481
	≥9 years	14 (15.1)	4 (28.6)	9 (64.3)	1 (7.1)	

Baseline cognitive score for each group were listed in Table 2. After 12 weeks of intervention, this study found an improvement in the cognitive domains of memory, verbal fluency, and learning process in group A, and an improvement in visuospatial, memory, and verbal fluency cognitive domains in group B. Only improvement of memory function was found in the Control group. Improvement of the learning process only occurred in group A (see Table 4). It can be concluded that probiotic intervention can increase cognitive functions of memory, visuospatial, and verbal fluency (*p* < 0.05), with improvements in learning processes being more noticeable in intervention group A.

**Table 4:** Cognitive score differences between group A, B, and Control.

**Cognitive domain**	**Variable**	**Group A (*n =* 33)**			**Group B (*n =* 32)**			**Control group (*n =* 28)**		
		***n***	**Median (Min–Max)**	***p*-value**	**n**	**Median (Min–Max)**	***p*-value**	***n***	**Median (Min–Max)**	***p*-value**
Executive function and visuospatial	CDT pre	33	0.00 (0–2)	0.083	32	0.00 (0–2)	0.008*	28	0.00 (0–2)	0.257
	CDT post	33	0.00 (0–2)		32	2.00 (0–2)		28	2.00 (0–2)	
Memory	WLMIR 1 pre	33	2.00 (0–5)	0.099	32	2.00 (0–6)	0.002*	28	3.00 (0–5)	0.003*
	WLMIR 1 post	33	3.00 (0–6)		32	3.50 (0–6)		28	4.00 (1–7)	
	WLMIR 3 pre	33	4.00 (0–8)	0.000*	32	5.00 (0–8)	0.017*	28	5.00 (3–9)	0.017*
	WLMIR 3 post	33	6.00 (2–9)		32	6.00 (0–10)		28	6.00 (3–10)	
	Learning process pre	33	2.00 (-2–4)	0.006*	32	2.00 (-3–6)	0.641	28	3.00 (0–5)	0.946
	Learning process post	33	3.00 (-2–7)		32	2.50 (0–6)		28	3.00 (0–5)	
Language	Verbal fluency pre	33	10.00 (5–18)	0.034	32	11.00 (4–24)	0.000*	28	12.00 (3–20)	0.436
	Verbal fluency post	33	12.00 (4–18)		32	14.50 (6–25)		28	11.50 (0–25)	

*CDT, Clock Drawing Test; WLMIR, Word List Memory Immediate Recall*.

^*^p < 0.05 using Wilcoxon Test.

## Discussion

### Probiotics and Cognitive Function

Most of the subjects were aged 65 years and over (77.4%), female (64.5%), and had an educational background of below 9 years (84.9%). The sociodemographic distribution of participants in this study is similar to previous studies (Handajani et al., 2020; Suriastini et al., 2020) that enrolled subjects from communities in Indonesia. The participation of subjects in these studies, and likewise in ours, is influenced by employment status (resulting in a higher involvement of women than men), and by the majority of Indonesia's elderly population having a poor level of education (63%) (Handajani et al., 2020; Suriastini et al., 2020; Statistik Penduduk Lanjut Usia, 2021). Results from this study reaffirm that the improvement in cognitive function when consuming tempeh is due to the content of microbiota and probiotics in tempeh. In a previous study, consumption of tempeh for 6 months was found to significantly increase cognitive function in the elderly population group with mild cognitive impairment (MCI) (Handajani et al., 2020). Animal studies found that the extract from tempeh can affect brain function through its role in the gastrointestinal system (Hamad et al., 2016; Kridawati et al., 2020). In a study comparing the administration of tempeh and soy milk for 28 days, Stephanie et al. (2018) found that tempeh consumption modulated gut microbiota, increasing amounts of *Bifidobacterium* and *A. muciniphila*.

Although tempeh comes from the fermentation of *Rhizopus oligosporus*, many other types of bacteria found in tempeh can be beneficial. In this study, the isolated and identified bacteria was *L. fermentum*. This is in accordance with the study done by Radita et al. (2017) where the dominant bacteria in tempeh was found to be *Lactobacillus* from the phylum *Firmicutes*, formed from the tempeh soaking process.

Several mechanisms explain the influence of the microbiota on cognitive function, occurring through the GBA, including neurological and endocrine pathways. Probiotics that enter the digestive system will affect neurological pathways such as the vagus nerve and neurotransmitter activities in the gastrointestinal tract, including GABA, serotonin, melatonin, and acetylcholine, among others, activating catecholamines. The endocrine pathway affects GBA through the activation of enteroendocrine cells. Probiotics consumed produce metabolites such as short-chain fatty acids (SCFA) for the synthesis of serotonin, which contributes to metabolic pathways in the brain. A disturbance in the environment of the gut microbiota can induce cognitive impairment (Appleton, 2018).

In addition, probiotics also contain peptidoglycans, a unique component that helps regulate the immune system among its many roles and also found in brain tissue due to systemic translocation. Through interactions with specific receptors, such as peptidoglycan recognition protein (PGRP) and Nod-like receptors, peptidoglycans affect motor, socio-emotional, and cognitive development processes (Tosoni et al., 2019). *L. fermentum*, itself, can reduce neuroinflammation and memory impairment caused by LPS. In addition to the production of neuromodulators, memory enhancement is also caused by the administration of *L. fermentum*-containing milk which inhibits AChE activity (Musa et al., 2017).

Kobayashi et al. (2019a,b) showed a significant increase in cognitive function of delayed recall memory in the subgroup with cognitive deficits, where the intervention had a greater impact on subjects with cognitive deficits. Research by Beltagy et al. (2021) also demonstrated increased concentrations of acetylcholine, dopamine, serotonin, and anti-oxidants in Alzheimer's disease, as well as increased ATP1A1 activity in the hippocampus, from probiotic supplementation. Another study assessed changes in the composite z score of three measures of memory and attention during 12 weeks of administration of *Lactobacillus plantarum* mixed with fermented soybean powder and found significant improvements in memory and attention scores (Hwang et al., 2019).

Athari Nik Azm et al. (2018) conducted a study by giving probiotic powder to Wistar rats every day for 8 weeks, then rats were induced to develop Alzheimer's with amyloid (Aβ1-42). Learning and memory behavior were tested using the Morris Water Maze Test. The rats' drinking water was mixed with probiotic powder *Lactobacillus acidophilus, L. fermentum, Bifidobacterium lactis*, and *Bifidobacterium longum*. The time and distance required for the Alzheimer's-probiotic (AP) group in the test decreased significantly compared to the Alzheimer's (Aβ) group. Administration of probiotics in the AP group could prevent learning and memory decline due to an increase in brain-derived neurotrophic factor (BDNF) expression and GABA production (Athari Nik Azm et al., 2018).

One study also reported significant improvements in attention and memory domains in the probiotic group, with significantly greater attention scores than the placebo group after the intervention. However, some improvements were also seen in the learning and recall subtests in the placebo group, which the authors acknowledge can represent a learning effect across the test (Ohsawa et al., 2018).

Improvements in cognitive assessment results may be associated with improvements in other aspects, such as physical performance, which is a known predictor of cognitive impairment (Kamo et al., 2018; Ogawa et al., 2018).

Our study found that probiotic intervention in group A, which had lower overall memory scores before the intervention (WLMIR 3 score pre-test), had a more significant effect on improving the learning process. Learning process (assessment of recurring memory, which was the substraction between WLMIR 3 and WLMIR 1, comparing post and pre-test) is a better assessment to evaluate improvement of learning and new memory, compared to single assessment (WLMIR 1).

### Mechanism of Action

This study succeeded in detecting three LAB isolates that had gene encoding glutamate decarboxylase (*gad*), an enzyme involved in synthesizing GABA. The study conducted by Gao et al. proved that GABA concentrations decreased with age after reaching adulthood. GABA concentrations in the frontal cortex are estimated to decrease by as much as 5% per decade with age. Decreased concentrations of GABA in the frontal cortex may cause cognitive decline because the frontal cortex plays an essential role in cognitive function (Gao et al., 2013).

Spatial and temporal memory is associated with neuronal oscillatory activity in the hippocampus, where the N-methyl-D-aspartate (NMDA)-type glutamate receptors on neurons play a role in modulating memory. In addition to NMDA receptors, 5-GABAA receptors at the base of dendrites receive excitatory input. Both receptors are complemented in controlling signal transduction in hippocampal cells (Möhler, 2009).

In several studies, LAB has been shown to increase elderly cognitive function by producing neurotransmitters and neuromodulators, reducing neuroinflammation, inhibiting acetylcholinesterase (AChE), and increasing BDNF expression (Akbari et al., 2016; Musa et al., 2017; Athari Nik Azm et al., 2018).

Bacterial species can produce large amounts of neurotransmitters, including GABA, dopamine, serotonin, and norepinephrine, as well as increase the availability of precursors such as tryptophan (Holzer and Farzi, 2014; Yano et al., 2015). Probiotics can also increase the availability of neuroactive compounds indirectly by stimulating metabolites through biosynthesis (Yano et al., 2015). Studies suggest that probiotic-induced changes in the gut were likely to cause functional changes in the brain, including behavioral shifts. However, the precise mechanism by which changes in gut metabolites mediate these neurochemical changes remains unclear. In addition to altered neurotransmitter production, it is thought that probiotics may influence the production of other bacterial-derived metabolites, particularly SCFA, which are thought to be involved in GBA communication (Dalile et al., 2019; Silva et al., 2020). *In vitro* models have shown an increase in SCFA (particularly acetate, butyrate and propionate) as a result of probiotic bacteria (Nagpal et al., 2018). Moreover, Wang et al. (2018) conducted a trial among young, adult, and older groups with *L. plantarum* and found that fecal acetate and propionate levels increased significantly in all three age groups, and slowly decreased to near baseline levels after supplementation stopped. Probiotics can also increase the availability of neuroactive compounds indirectly by stimulating metabolites for biosynthesis.

Probiotics have been associated with increased gut barrier integrity and reduced permeability; this was thought to occur due to increased mucin expression and tight-junction stability that protects the epithelial barrier (Stoidis et al., 2011; Hemert et al., 2013). Consequently, probiotic interventions can reduce endotoxemia and inflammation levels.

In addition, probiotics can attenuate the deleterious effects of pro-inflammatory cytokines on the gut barrier by reducing pro-inflammatory and enhancing anti-inflammatory responses. Studies have shown that chronic supplementation with *L. salivarius* has been associated with significant reductions in serum concentrations of inflammatory markers such as high sensitivity C-reactive protein (hs-CRP), interleukin (IL)-6, IL-1b, and TNF-α (Rajkumar et al., 2015).

This study is the random sampling without blinding and the viability test of probiotics after solubilization in the solution has not been conducted and might become a limitation of this study. In the future, it is important to conduct study with subjects with severe cognitive conditions such as dementia with a longer intervention duration.

## Conclusion

Administration of probiotics derived from *L. fermentum* at concentrations of 10^8^ CFU/mL and 10^7^ CFU/mL increased the cognitive domains of memory, language, and visuospatial function. However, probiotic supplementation with a 10^8^ CFU/mL concentration was better at improving the learning process. This study succeeded in detecting Lactic Acid Bacterial isolates *L. fermentum* that had a gene encoding glutamate decarboxylase (gad) which is involved in the synthesis of GABA, known to play an essential role in cognitive function.

## Data Availability Statement

The original contributions presented in the study are included in the article/supplementary material, further inquiries can be directed to the corresponding author/s.

## Ethics Statement

This research was approved by the Ethics Committee of Atma Jaya Unika FKIK NO: 10/07/KEP/-FKUAJ/2019. The patients/participants provided their written informed consent to participate in this study.

## Author Contributions

Study concept and design: YH, YT, and YY. Design of the studies and data acquisition: YH, YT, and NW. Analysis and interpretation of data: SPS and VL. Drafting the manuscript: YH, YT, YY, AS, GAMC, SPS, VL, and NW. Critical revision of the manuscript: YH, YT, YY, and AS. All authors contributed to the article and approved the submitted version.

## Funding

This study was funded by the Kementerian Riset dan Pendidikan Tinggi Republik Indonesia Grant (ID 17/AKM/PNT/2019, March 27, 2019). The funders had no role in study design, data collection and analysis, decision to publish, or manuscript preparation.

## Author Disclaimer

The content is solely the responsibility of the authors and does not necessarily represent the funders.

## Conflict of Interest

The authors declare that the research was conducted in the absence of any commercial or financial relationships that could be construed as a potential conflict of interest.

## Publisher's Note

All claims expressed in this article are solely those of the authors and do not necessarily represent those of their affiliated organizations, or those of the publisher, the editors and the reviewers. Any product that may be evaluated in this article, or claim that may be made by its manufacturer, is not guaranteed or endorsed by the publisher.
